# Use of the Land Snail *Helix aspersa* as Sentinel Organism for Monitoring Ecotoxicologic Effects of Urban Pollution: An Integrated Approach

**DOI:** 10.1289/ehp.8397

**Published:** 2005-09-20

**Authors:** Francesco Regoli, Stefania Gorbi, Daniele Fattorini, Sara Tedesco, Alessandra Notti, Nicola Machella, Raffaella Bocchetti, Maura Benedetti, Francesco Piva

**Affiliations:** Istituto di Biologia e Genetica, Università Politecnica delle Marche, Ancona, Italy

**Keywords:** atmospheric pollutants, bioindicators, biomarkers, DNA integrity, lysosomes, metallothioneins, oxidative stress, peroxisomes, polycyclic aromatic hydrocarbons, trace metals

## Abstract

Atmospheric pollution from vehicular traffic is a matter of growing interest, often leading to temporary restrictions in urban areas. Although guidelines indicate limits for several parameters, the real toxicologic impacts remain largely unexplored in field conditions. In this study our aim was to validate an ecotoxicologic approach to evaluate both bioaccumulation and toxicologic effects caused by airborne pollutants. Specimens of the land snail *Helix aspersa* were caged in five sites in the urban area of Ancona, Italy. After 4 weeks, trace metals (cadmium, chromium, copper, iron, manganese, nickel, lead, and zinc) and polycyclic aromatic hydrocarbons (PAHs) were measured and these data integrated with the analyses of molecular and biochemical responses. Such biomarkers reflected the induction of detoxification pathways or the onset of cellular toxicity caused by pollutants. Biomarkers that correlated with contaminant accumulation included levels of metallothioneins, activity of biotransformation enzymes (ethoxyresorufin *O*-deethylase, ethoxycoumarin *O*-deethylase), and peroxisomal proliferation. More general responses were investigated as oxidative stress variations, including efficiency of antioxidant defenses (catalase, glutathione reductase, glutathione *S*-transferases, glutathione peroxidases, and total glutathione) and total oxyradical scavenging capacity toward peroxyl and hydroxyl radicals, onset of cellular damages (i.e., lysosomal destabilization), and loss of DNA integrity. Results revealed a marked accumulation of metals and PAHs in digestive tissues of organisms maintained in more traffic-congested sites. The contemporary appearance of several alterations confirmed the cellular reactivity of these chemicals with toxicologic effects of potential concern for human health. The overall results of this exploratory study suggest the utility of *H. aspersa* as a sentinel organism for biomonitoring the biologic impact of atmospheric pollution in urban areas.

Increased vehicular traffic and emissions are major contributors to air pollution and a matter of growing importance in many city centers [World Health Organization (WHO) 2000]. Human and ecotoxicologic risks range from asthmatic, respiratory, and cardiovascular problems to long-term effects caused by carcinogenic and mutagenic properties of many chemicals associated in complex mixtures, with overall biologic effects difficult to predict (Maynard 2004).

Normative limits and international guidelines indicate the maximum levels for a number of individual pollutants in air samples. Severe traffic restrictions were imposed recently in many Italian cities after values for particulate matter ≤ 10 μm in aerodynamic diameter (PM_10_) were exceeded, resulting in a public discussion on such political decisions. Municipalities often rely on automatic monitoring stations for their air quality programs, which is useful for defining both short- and long-term variations. However, this approach is somewhat limited. The relatively elevated costs for installation and maintenance sometimes preclude the detailed monitoring of large urban areas. In addition, automatic stations generally analyze only a set of parameters (e.g., PM_10_, ozone, benzene, carbonic monoxide, sulfur oxides), whereas other factors primarily involved in risk disease, such as PM_2.5_ (PM ≤ 2.5 μm in aerodynamic diameter), polycyclic aromatic hydrocarbons (PAHs), or metals are not detected. Most important, instrumental analyses do not account for interactions among different chemicals co-occurring in complex mixtures, and the association of these data with the onset of deleterious biologic effects are debated and controversial (Gamble 1998; Mainard 2004).

In contrast to automatic monitoring techniques, the study of bioindicator organisms can reveal the biologic impact of pollution over a geographical and temporal scale, depending on the selected species and approach. Mosses and lichens have been recognized as suitable biomonitors or bio-accumulators for air pollution (Bargagli 1998; Bargagli et al. 2002; Cislaghi and Nimis 1997; Wolterbeek 2000), and terrestrial invertebrates are used for monitoring air and soils (Dallinger 1994).

Among terrestrial invertebrates, the gastropods *Helix* spp. have the capability to accumulate different classes of chemicals and serve as pertinent species for monitoring trace metals, agrochemicals, urban pollution, and electromagnetic exposure (Beeby and Richmond 2002, 2003; Berger and Dallinger 1993; Gomot de Vaufleury and Pihan 2000; Regoli et al. 2005; Snyman et al. 2000; Viard et al. 2004). Other biologic effects have also been described, including growth inhibition, impairment of reproductive capacity, and induction of metallothioneins (MT); specific proteins are involved in metal homeostasis and detoxification (Dallinger 1996; Gomot-de Vaufleury and Kerhoas 2000). Pollutants accumulated through different routes are transported by blood cells to the digestive gland, which also represents the main target organ for metabolic and detoxification processes (Beeby and Richmond 2002; Regoli et al. 2005).

The main objective of the present study was to develop an integrated ecotoxicologic approach with the land snail *Helix aspersa* for monitoring both accumulation and toxicologic effects caused by urban pollutants, including vehicular exhausts and other chemicals such as those associated with tire manufacturing, which can be transported by PM from the road surface. The use of sentinel species is of particular interest to assess biologic reactivity of such complex mixtures that are difficult to characterize on a chemical basis. Analyses of individual analytes in abiotic matrices do not necessarily relate to their bioavailability and do not evaluate synergistic or cumulative effects caused by various classes of chemicals. In this study, organisms were caged in different sites within the city of Ancona, Italy, and analyzed after 4 weeks for the trace metals and PAHs chosen as model chemicals potentially associated with urban pollution. We evaluated the biologic significance of these data using the assessment of a wide panel of molecular–biochemical alterations (biomarkers) reflecting both the induction of specific metabolic/detoxification pathways and the early onset of cellular damages caused by different classes of pollutants or chemical mixtures. Among specific responses, induction of MT, cytochrome P450, and peroxisomal proliferation were selected for metals and organic aromatic pollutants. Although the biotransformation pathway of cytochrome P450 is often not consistent in invertebrates (Livingstone et al. 2000), there is some evidence of its involvement in the metabolism of xenobiotics in gastropods (Ismert et al. 2002). Proliferation of peroxisomes has also been documented as a toxicologic effect of several chemicals in both vertebrate and invertebrate models (Cancio and Cajaraville 2000; Lock et al. 1989). A general pathway of toxicity for several pollutants is mediated by the enhancement of intracellular reactive oxygen species (ROS), which often modulate the occurrence of cell damage (Regoli et al. 2002, 2003). In the present study, we measured variations of antioxidant defenses as biomarkers of contaminant-mediated pro-oxidant challenge. The overall susceptibility to oxidative stress conditions was also assessed by the total oxyradical scavenging capacity (TOSC) assay, which quantifies the capability to neutralize specific ROS such as peroxyl radicals (ROO•) and hydroxyl radicals (HO•) (Gorbi and Regoli 2003). To further investigate pollutant-mediated oxidative toxicity, we estimated lysosomal membrane stability and loss of DNA integrity as typical targets of environmental contaminants, which act through direct mechanisms or enhanced oxyradical formation (Moore et al. 2004; Regoli 2000; Regoli et al. 2004).

The use of these cellular biomarkers is also of potential interest for assessing the impact of air pollution on human health. A large proportion of PM originates from mobile sources and includes both aromatic hydrocarbons and trace metals (Shi et al. 2001). Several epidemiologic and laboratory investigations support the evidence that these chemicals induce inflammatory responses through enhanced formation of ROS and other cellular mechanisms modulated by antioxidant variations and oxidative injuries (Sioutas et al. 2005). Recent studies also revealed a higher incidence of genotoxic damages in traffic police and populations exposed to moderate levels of PAHs in urban areas (Kyrtopoulos et al. 2001; Maffei et al. 2005).

We expected the overall results of this study on *H. aspersa* to provide useful indications on the biologic reactivity and toxicologic effects of atmospheric pollutants in field conditions, to assess the validity of *Helix* spp. as a model for human disease outcomes, and investigate the possibility of integrating a multimarker ecotoxicologic approach in air quality programs in urban areas.

## Materials and Methods

### Experimental design.

This study was carried out in the city of Ancona in central Italy, where five locations were chosen for caging experiments. An extraurban area was the reference (site 1); the other stations were selected according to characteristics of daily vehicular traffic. At site 2, a relatively small and one-way street, snails were caged 200 m past a traffic light and thus were exposed to moving cars. More elevated and slower traffic flows characterized site 3, close to the entrance of the university complex, and to a greater extent, sites 4 and 5, located before a traffic light on a large road and in proximity to a tunnel, respectively. Based on estimates carried out during the peak time, the Office for Public Works and Traffic of Ancona Municipality indicated traffic intensities of 25,900 vehicles/hr for the whole urban area; between 4,000 and 3,000 vehicles/hr at sites 4 and 5; between 1,200 and 1,800 vehicles/hr at site 3; between 600 and 1,200 vehicles/hr at site 2; and < 600 vehicles/hr at site 1 (Piano Generale del Traffico Urbano 2005). Cars were the dominant vehicles in all the sites, although an elevated number of mopeds (some hundreds) also passed through site 3. No other sources of pollutants were noted at the investigated sites.

Gastropods *H. aspersa* (4–6 g total weight) were purchased from a local farm, divided into groups of 50 specimens, and settled in plastic cages (50 × 40 × 20 cm) excluding a direct contact with soil. At least two cages were deployed in May 2004 within approximately 1 m from the road margin at each location. Daily, transplanted snails were fed carrots and moistened to prevent the occurrence of a dormancy state. After 4 weeks of exposure, the mortality rate appeared < 10% in all the sites, and the snails were recovered and sacrificed. Digestive glands were rapidly dissected out, frozen in liquid nitrogen, and stored −80°C. Hematocytes were withdrawn from the hemocel cavity and immediately processed for assessment of lysosomal membrane stability and DNA integrity. Animals were treated humanely and with regard for alleviation of suffering.

### Chemical analyses.

We measured trace metals and PAHs in composite pools of digestive glands dissected from 20 snails (five samples, each constituted by tissues of four specimens). For trace metals, tissues were dried at 60°C until they reached a constant weight, and approximately 0.5 g dried samples were digested under pressure with 5 mL nitric acid and 1 mL hydrogen peroxide in a microwave digestor system (Microwave Laboratory System; Milestone, Shelton, CT, USA). Quality assurance and quality control were tested by processing blank samples and standard reference material (SRM; mussel tissue SRM 2977; National Institute of Standards and Technology, Gaithersburg, MD, USA). Metals (cadmium, chromium, copper, iron, manganese, nickel, lead, and zinc) were analyzed by atomic absorption spectrophotometry with electrothermal atomization (SpectrAA 300 Zeeman, Varian, Mulgrave, VIC, Australia) and flame atomization (Varian SpectrAA 220FS, Varian) and expressed as micrograms per gram dry weight. When necessary, we applied the standard addition technique for resolution of matrix effects, and a palladium solution (1 mg/mL, 10% nitric acid, 10% citric acid) was added as chemical matrix modifier. The concentrations obtained for the SRM were always within the 95% confidence interval of certified values.

For PAHs, about 1 g digestive tissues (wet weight) were extracted in 5 mL 0.5 M potassium hydroxide in methanol with a microwave (150 W for 10 min). Samples were centrifuged at 1,000 × *g* for 5 min. Methanolic solutions were concentrated in a SpeedVac (RC1009; Jouan, Nantes, France) and purified with solid-phase extraction (Octadecyl C18, 500 mg × 6 mL, Bakerbond; Mallinckrodt Baker, Phillipsburg, NJ, USA). A final volume of 1 mL was recovered with acetonitrile, and HPLC analyses were carried out using a water-acetonitrile gradient and fluorimetric detection. Individual PAHs were identified by the retention time of appropriate pure standard solutions, and the quality assurance/quality control were tested by processing blank and references samples (mussel tissues SRM 2977, NIST). The concentrations obtained for the SRM were always within the 95% confidence interval of certified value. The water content in tissues was determined during preparation of samples for metal analysis and used to normalize PAH concentration (micrograms per gram) to dry weight.

### Biochemical analyses.

These determinations were carried out in composite pools of digestive glands dissected from 20 snails (five samples, each constituted by tissues of four specimens). For the analysis of MT, samples were homogenized [1:3 weight/volume (wt/vol)] in 20 mM Tris-HCl buffer (pH 8.6), 0.5 M sucrose, 0.006 mM phenylmethylsulfonyl fluoride (PMSF) and 0.01 % β-mercaptoethanol. After acidic ethanol/chloroform fractionation of the tissue homogenate, MT were quantified by a spectrophotometric assay using reduced glutathione (GSH) as standard (Viarengo et al. 1997).

We measured ethoxyresorufin *O*-deethylase (EROD) and ethoxycoumarin *O*-deethylase (ECOD) activities after homogenization (1:5 wt/vol) in 0.1 M K-phosphate buffer (pH 7.5), 0.15 M KCl, and 1 mM EDTA. After centrifugation at 12,000 × *g* for 15 min (Regoli et al. 2003), 250 μM β-nicotinamide adenine dinucleotide (NADPH) was added to S9 aliquots in 0.1 M K-phosphate buffer (pH 7.4) containing 7-ethoxyresorufin (4 μM in dimethyl sulfoxide) or 7-ethoxycoumarin (50 μM in ethanol). Reactions were stopped after 5 min, and blank values were subtracted. Fluorescence samples were quantified by a calibration curve with resorufin or 7-hydroxy-coumarin standards, using 535 or 380 nm (excitation wavelength) and 585 or 460 nm (emission wavelength), respectively.

We analyzed peroxisomal proliferation by the activity of acyl-coenzyme A oxidase (AOX) in samples homogenized (1:5 wt/vol) in 1 mM NaHCO_3_, 1 mM EDTA, 0.1% ethanol, and 0.01% Triton X-100 and then centrifuged at 500 × *g* for 15 min at 4°C. AOX was spectrophotometrically measured in supernatants according to Small et al. (1985). The H_2_O_2_ production was followed at 502 nm by the oxidation of dichlorofluorescein-diacetate catalyzed by an exogenous horseradish peroxidase (HRP). A final volume of 1 mL contained 0.5 M K-phosphate buffer (pH 7.4), 2.2 mM dichlorofluorescein-diacetate (DFA-DA), 40 μM sodium azide, 0.01 % Triton X-100, and 1.2 U/mL HRP; 30 μM palmytoil-CoA was added as substrate for AOX after a pre-incubation of 5 min in the dark.

Enzymatic antioxidants were measured in samples homogenized (1:5 wt/vol) in 100 mM Tris-HCl buffer (pH 8.0), 0.1 mM PMSF, 0.008 trypsin inhibitor units/mL aprotinin, 1 μg/mL leupeptin, 0.5 μg/mL pepstatin, and 0.6% NaCl and centrifuged at 100,000 × *g* for 1 hr at 4°C to obtain cytosolic fractions. Spectrophotometric measurements were carried out as described elsewhere (Regoli et al. 2004). Catalase was quantified by the decrease in absorbance at 240 nm due to H_2_O_2_ consumption. Glutathione reductase (GR) activity was followed by the oxidation of NADPH at 340 nm during the reduction of oxidized glutathione (GSSG). Glutathione peroxidases (GPx) were measured at 340 nm in a coupled enzyme system where cumene hydroperoxide is used as substrate for the sum of Se-dependent and Se-independent forms and NADPH is consumed by GR to convert the formed GSSG to its reduced form. Glutathione *S*-transferases (GST) were determined at 340 nm using 1-chloro-2,4-dinitrobenzene as substrate. Total glutathione was analyzed after homogenization (1:5 wt/vol) of tissues in 5% sulfosalicilic acid with 4 mM EDTA. Samples were maintained for 45 min on ice and centrifuged at 37,000 × *g* for 45 min. The resulting supernatants were assayed by following the GR-catalyzed reaction of GSH with 5,5′-dithiobis-2-nitrobenzoic acid and comparing this rate with a standard GSH curve.

TOSC was measured in samples homogenized as described above for the enzymatic antioxidants, without adding PMSF to the buffer. The TOSC assay quantifies the capability of cellular antioxidants to inhibit the oxidation of 0.2 mM α-keto-γ-methiolbutyric acid to ethylene gas in the presence of different forms of oxyradicals artificially generated at a constant rate (Regoli and Winston 1999; Winston et al. 1998). ROO• and HO• were generated by the thermal homolysis of 20 mM 2-2′-azo-bis-(2-methylpropionamidine)-dihydrochloride and from an Fe ascorbate Fenton reaction (Regoli and Winston 1999), respectively. TOSC values were quantified from the following equation:


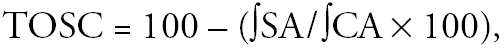


where ∫SA and ∫CA are the areas integrated under the kinetic curves for sample (SA) and (CA) reactions, respectively (Winston et al. 1998). TOSC values were normalized to content of proteins, measured in both S9 and cytosolic fractions with the Lowry method and bovine serum albumin as standard.

### Neutral red retention time assay.

Lysosomal membrane stability was measured in freely circulating hematocytes by the neutral red retention time (NRRT) assay, which quantifies the capability of these organelles to retain the vital dye (Regoli 2000; Snyman et al. 2000). Hemolymph was withdrawn from the visceral hemocel of 10 individual snails and incubated on a microscope slide with a neutral red working solution as previously described (Regoli et al. 2005). Hematocytes were observed under a light microscope at 2-min intervals, and only the most abundant cell type, namely, the smaller hyaline and agranular hematocytes with pseudopodia, were considered. The NRRT was calculated as the time at which ≥ 50% of the counted cells presented reddish cytosols after the leakage of the dye from lysosomes.

### Single-cell gel electrophoresis.

We performed the comet assay on hematocytes freshly collected from 10 snails; the cells were diluted in Ca^2+^- and Mg^2+^-free buffers (20 mM HEPES, 120 mM NaCl, 5 mM KCl, 10 mM EDTA), and spun at 1,000 rpm for 1 min at 4°C. Detailed procedures for sample preparation and comet assay conditions have been described elsewhere (Regoli et al. 2005). After electrophoresis, slides were stained with SYBR green 1X (Molecular Probes, Leiden, The Netherlands) and observed under a fluorescence microscope (200× magnification, Eclipse E-600, Nikon, Kawasaki, Japan). At least 100 randomly selected cells from each slide and two replicates per sample were counted and classified in five classes of damage according to the length of DNA migration and the relative proportion of head/tail fluorescence (Collins 2002), as follows:

- Class 1: intact DNA without migrated fragments
- Class 2: dense nucleus with slight migration and a small tail
- Class 3: tails have separated from the nucleus, with a weaker fluorescence
- Class 4: clear tails that may reach full length
- Class 5: nucleus appears small and completely separated from the tail.

Comet results are given as percentage distribution of cells within the various classes. We summarized these data in a synthetic index of total damage (TD) calculated according to the following equation:


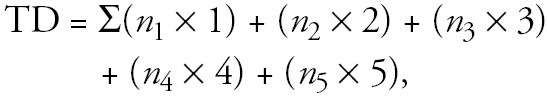


where *n*_1_, *n*_2_, *n*_3_, *n*_4_, and *n*_5_ indicate the percentage of cells within each of five classes of damage. Thus, TD ranges between 100 and 500, corresponding to the totality of cells in class 1 or class 5, the lowest and highest level, respectively, of DNA damage.

### Statistical analyses.

We performed statistical analyses using Statistica Software (version 6.0; Stat Soft, Tulsa, OK, USA). Chemical and biochemical parameters in snails from different sites were compared by one-way analysis of variance (ANOVA). The homogeneity of variance was analyzed by Cochran *C*, and post hoc tests (Newman-Keuls) were used to discriminate between means of values. The non-parametric Kruskal-Wallis test was applied to the results of the comet assay for comparing the distribution of cells within five classes of damage.

We used multivariate statistical analysis [principal component analysis (PCA)] to investigate correlations between the different variables.

## Results

Metal concentrations in the digestive gland of *H. aspersa* caged in May 2004 in different urban sites are reported in Table 1. A marked increase in Cr, Cu, Fe, Pb, Mn, Ni, and Zn was evident at site 5 and, with a few differences, at site 4. Compared with reference, the accumulation of metals was still significant at site 3 and at site 2, to a lesser extent, with values (especially for Pb and Cu) considerably lower than in other sites.

Higher concentrations of PAHs were also measured in caged snails with low molecular weight (lmw) hydrocarbons (i.e., naphthalene and fluorene) always prevailing over high molecular weight (hmw) congeners (i.e., fluoranthene, pyrene, benzo[*a*]anthracene, benzo-[*b*]fluoranthene, benzo[*k*]fluoranthene). Values of total PAHs increased from sites 2 and 3 to sites 4 and 5 (Table 1), but organisms at site 3 showed an elevated accumulation of pyrene and fluorene. Preliminary results from chemical data revealed sites 5, 4, and 3 as the most impacted, with the following approximate order of bioavailable pollutants for various urban areas: site 5 ≥ site 4 > site 3 >> site 2 ≥ site 1.

Variations in biochemical and cellular bio-markers are summarized in Table 2. Results on MT confirmed those on metals bio-accumulation, with levels significantly increasing from site 2 to the more traffic-congested sites (4 and 5). The activity of AOX revealed peroxisomal proliferation in organisms from sites 4 and 5 to a greater extent than in those at site 3. Cytochrome P450 assessed as EROD did not exhibit any change, whereas ECOD increased in specimens from sites 3 and 5.

Antioxidant responses showed different patterns of variations (Table 2). Catalase and GR were induced at sites 3, 4, and 5. Snails caged at site 5 exhibited significantly lower values for GST and a trend toward higher activities for GPx. The TOSC assay demonstrated an increased antioxidant efficiency in snails exposed in more traffic-congested sites (Table 2), with higher TOSC values toward both ROO• and HO•.

**Figure 1:**
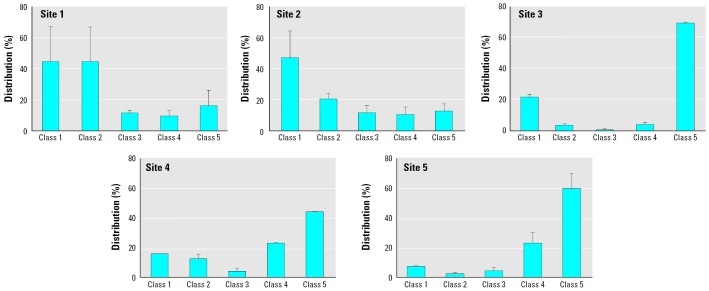
Loss of DNA integrity in snails caged in urban sites, expressed as the percentage distribution of cells within the five classes of DNA damage (mean ± SD; *n* = 5/group).

The lysosomal membrane stability was not compromised in snails from sites 1 and 3, whereas a significant destabilization was measured at sites 2, 4, and 5 (Table 2). The pattern of DNA damage was revealed by the comet assay, with a clear increase of percentage distribution of cells in classes 4 and 5 for snails caged at sites 3, 4, and 5 (Figure 1). These results were confirmed by the values of TD significantly higher in snails at more-impacted sites (Table 2).

From the PCA analysis, the first two axes explained 82% of the variance (Table 3). The factor loading showed that within the first axis, concentrations of Cu, Cr, Fe, Mn, Ni, Pb, Zn, naphthalene, fluorene, anthracene, pyrene, total lmw PAHs, total hmw PAHs, and total PAHs were positively correlated with the levels of MT; activity of AOX, ECOD, catalase, GR, TOSC toward ROO• and HO•; and onset of total DNA damage, whereas the same parameters were negatively associated with NRRT of lysosomes. In axis 2, positive associations were obtained for Cd, GPx, and levels of total glutathione and negative for Fe, phenanthrene, AOX, and GST.

**Figure 2:**
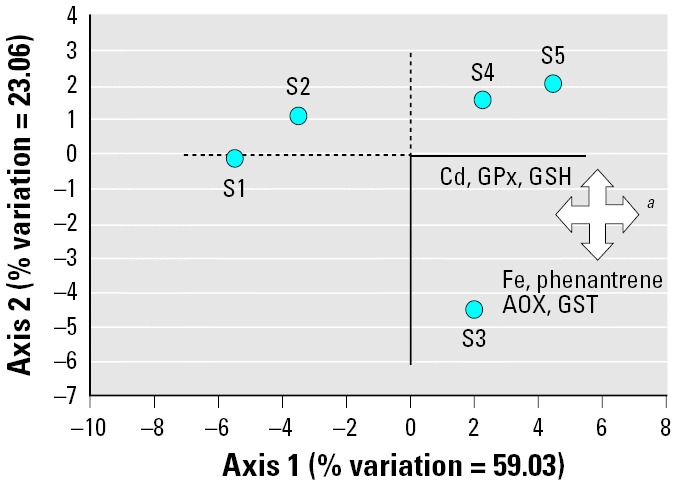
PCA results and separation of sites (S1, S2, S3, S4, and S5) on the basis of chemical residues and biologic parameters associated with axis 1 and axis 2 (see also Table 3). ***^a^***Cu, Pb, Cr, Ni, Mn, Zn, naphthalene, fluorene, anthracene, pyrene, lmw PAHs, hmw PAHs, total PAHs, MT, AOX, ECOD, catalase, GR, TOSC-ROO•, TOSC-HO•, DNA TD.

The ordination plot (Figure 2) confirmed the marked separation of sites 1 and 2 from sites 3, 4, and 5 on the basis of chemical residues and biologic parameters associated with axis 1. Snails at site 3 were further differentiated from those at sites 4 and 5 by the higher concentrations of Fe and phenanthrene, the more elevated activities of AOX and GST, and the reduced values for GPx and total glutathione (despite the fact that these latter parameters did not significantly change according to ANOVA).

## Discussion

These results demonstrate the possibility of an ecotoxicologic approach for assessing the biologic impact and risks from airborne and vehicular pollutants in urban areas. The use of caged snails might represent an improvement to actual monitoring techniques because the method is relatively cheap, easy to perform, and allows an active translocation procedure to investigate selected sites even in the absence of native organisms. In addition, the biologic significance of the results presented here is important both in terms of accumulated chemicals and appearance of toxicologic responses. Bioindicator organisms provide a time-integrated assessment of environmental quality reflecting the exposure over a 4-week translocation period, and thus are less affected by daily or even hourly fluctuations of chemical parameters.

Because we aimed to validate a protocol rather to monitor the urban area of Ancona, we selected a limited number of sites on the basis of vehicular traffic characteristics, and only one seasonal period was investigated. Overall results revealed marked effects in snails caged at various locations, that is, in the different accumulation of metals, which confirmed *H. aspersa* as a suitable bioindicator for these environmental pollutants (Beeby and Richmond 2002; Dallinger 1994). The digestive gland was the main target organ, with concentrations generally 5- to 10-fold higher than those measured in foot and lung (not shown). The uptake of contaminants in digestive tissues was not surprising (Beeby and Richmond 2003; Gomot de Vaufleury and Pihan 2000) and suggested that deposition and ingestion through PM was the main exposure route for such contaminants. The range of intersample variability for considered analytes was within expected values, based on other studies of chemical accumulation in terrestrial and marine invertebrates (Beeby and Richmond 2002; Berger and Dallinger 1993; Dallinger 1994; Gomot de Vaufleury and Pihan 2000; Regoli et al. 2004). Analysis of a minimum of five samples (each including tissues of at least four organisms) can thus be recommended to minimize erroneous interpretation of data. Levels of metals in snails caged at site 1 were typical for unpolluted reference organisms (Beeby and Richmond 2002; Dallinger 1994), whereas several elements were strongly accumulated in more traffic-congested sites (e.g., Pb, Cu, Zn, Cr, Fe, Mn, and Ni). Worthy to note is the variation of Pb, with concentrations increasing from < 2 μg/g up to 80 μg/g in snails caged close to the tunnel. Despite the use of unleaded gasoline, obligatory in Italy since January 2001 and expected to improve atmospheric pollution (Viard et al. 2004), our results indicated that this metal might still represent an important contaminant in urban areas. A persistent role of soil particles as an additional exposure route for Pb in snail tissues could be hypothesized, considering that this element can remain in soils for several years after the conversion of a country to unleaded gasoline. The marked accumulation of metals was also reflected by more elevated content of PAHs in snails caged at sites 4 and 5 and, for pyrolitic combustion-derived congeners, also in organisms exposed at site 3.

One of the main objectives of this study was to demonstrate the suitability of a wide battery of cellular biomarkers for assessing the earliest responses to atmospheric pollutants and the onset of toxicologic alterations which might be of concern also for human health. The overall results from multivariate analysis confirmed the possibility to discriminate the most impacted sites (sites 3, 4, and 5), where accumulation of chemical residues in digestive gland of snails correlated with a large number of biologic alterations. The significant induction of MT in snails with higher concentrations of metals demonstrated that these elements were accumulated in a biologically active form. Two distinct MT isoforms have been characterized previously in the digestive gland of *Helix pomatia*, the Cu-MT principally involved in homeostasis of Cu, and the Cd-MT inducible by exposure to metals (Dallinger et al. 2004). Our data did not discriminate between the isoforms but further supported these proteins as an excellent biomarker of metals contamination in different field conditions (Dallinger 1996).

Among biologic effects caused by aromatic xenobiotics, proliferation of peroxisomes in mammalian systems appears to have a role in hepatic carcinogenesis (Lake 1995). Metabolism of peroxisomes and mechanism of responses are largely unknown in invertebrates, with limited data available only for some marine species (Cancio and Cajaraville 2000). This study provided the first characterization of AOX in *H. aspersa*, showing basal activities comparable to the bivalve *Mytilus galloprovincialis* (Cancio and Cajaraville 2000). The significant induction of AOX in more polluted sites would also indicate the responsiveness of peroxisomes to atmospheric pollutants. Future investigations at the molecular level should be carried out to clarify if any mechanistic relationship exists between accumulation of fluorene, phenanthrene, anthracene, and pyrene and the contemporary induction of both peroxisomal proliferation and GST as indicated by PCA analysis in snails from site 3. It is unknown why snails at site 3 showed higher levels of some contaminants and different biologic responses compared with sites 4 and 5. The possible influence of moped traffic can be only speculated.

Biotransformation of PAHs by cytochrome P450 is controversial in terrestrial invertebrates. Some evidence of benzo[*a*]pyrene metabolism has been shown in the earthworms *Lumbricus terrestris* and *Eisenia fetida*, where some isoforms were induced but others did not respond to PAHs (Lee 1998). In land snails, the digestive gland of *H. aspersa* exhibited a low (1.3- to 1.5-fold) but significant induction of either EROD or ECOD activity after exposure to a naphthalene-saturated atmosphere (Ismert et al. 2002). Our results confirmed the presence of cytochrome P450 activities in these gastropods but low levels of EROD, and the highly variable responses for ECOD did not support a clear role of biotransformation enzymes in metabolism of xenobiotics, nor their suitability as biomarkers for monitoring programs with *H. aspersa*.

Airborne pollutants cause a significant perturbation of the redox status, as indicated by the wide spectrum of oxidative parameters characterized in *H. aspersa*. Among these, catalase showed elevated basal activities approximately 5- to 10-fold greater than those typical of marine mollusks (Regoli et al. 2004; Regoli and Principato 1995), thus indicating an efficient protection toward H_2_O_2_, a potent oxidant and the main precursor of HO•(Regoli et al. 2004). Nonetheless, the significant increase of both catalase and GR reflects a varied pro-oxidant challenge in snails caged in more traffic-congested sites 3, 4, 5. Catalase has already been reported to be sensitive to chemical pollutants in aquatic bioindicators (Livingstone 2001; Regoli et al. 2003; Regoli and Principato 1995). Translocation experiments also demonstrated the possibility of biphasic variations, where initial counteracting responses might be followed by inhibition at longer exposure periods (Regoli et al. 2004; Regoli and Principato 1995). On the other hand, variations of GR modulate responsiveness of glutathione metabolism in invertebrates by increasing the capability to reconvert oxidized GSSG to the functionally active GSH (Regoli et al. 2002, 2003, 2004; Ringwood et al. 2004). Although laboratory exposures to naphthalene did not affect the activity of GR in *H. aspersa* (Ismert et al. 2002), our results confirmed the possibility of using this enzyme as a sensitive biomarker in field conditions.

Snails caged at site 5 exhibited a significant inhibition of GST, a multigene family that catalyzes both detoxification of organic compounds and antioxidant reactions through the reduction of hydroperoxides. Similarly to catalase, *H. aspersa* also showed elevated basal levels for these enzymes at least an order of magnitude above those commonly measured in the digestive gland of *M. galloprovincialis* (Regoli et al. 2004; Regoli and Principato 1995). Such elevated GST activities might explain the limited and fluctuating variations observed in various sites. However, contradictory results have often been described in field conditions, with increases, decreases, and transitory changes in these enzymes according to the intensity and duration of exposure (Regoli et al. 2003).

The effects on individual antioxidants were useful as sensitive warning signals of oxidative perturbation in most impacted sites (especially sites 3, 4, 5), but also confirmed complex interactions and responses that are not easy to predict. The overall biologic significance of these variations was better assessed by the measurement of TOSC, which summarizes in quantitative terms the susceptibility of a tissue to oxidative stress (Gorbi and Regoli 2003; Regoli et al. 2002; Regoli and Winston 1999). In the present study, increased TOSC values toward ROO• and HO• were measured in organisms caged at more traffic-congested locations (sites 3, 4, and 5), indicating that the higher pro-oxidant pressure and specific alterations of certain antioxidants (such as catalase, GR, GST) were reflected in a more integrated imbalance of oxyradical metabolism. A varied capability to neutralize ROS is of great value in assessing the biologic impact of pollutants because this alteration predicts the onset of other cellular damages in several animal models and in humans (Gorbi and Regoli 2003).

Our results confirmed the lysosomal membrane as a typical cellular target of chemical toxicity. Both lipophilic xenobiotics and metals alter the efficiency of membrane-bound proton pumps, increasing membrane permeability and eventually resulting in the loss of acid hydrolases into cytosol (Moore et al. 2004; Moore and Simpson 1992; Regoli 2000). These effects can be mediated by direct binding to the lysosomal membrane and indirectly by the enhanced formation of oxyradicals (Regoli 2000). The lysosomal compartment is highly developed in mollusks (Moore et al. 2004), and a significant reduction of NRRT was observed in snails caged in all urban areas, with the exception of site 3. Because of their elevated sensitivity, lysosomal biomarkers were confirmed as suitable tools for early detection of biologic disturbance, but they did not discriminate between sites with increasing levels of environmental pollutants. Normal values of NRRT measured in *H. aspersa* (approximately 30 min) were much lower than those typical for other invertebrates (90–120 min in *M. galloprovincialis*) (Regoli et al. 2004), but have been described as typical for this species (Snyman et al. 2000).

The evident accumulation of metals and PAHs and the general alterations of oxyradical metabolism were also reflected by genotoxic effects in snails exposed in more traffic-congested sites. Aromatic hydrocarbons have the potential to enhance oxyradical formation in invertebrates through redox cycling and impairment of cellular antioxidant systems (Livingstone 2001; Regoli et al. 2003). Similarly, an oxidative pathway for DNA damage has been documented for trace metals that can catalyze Fenton-like reactions, interact with –SH groups, and increase intracellular pro-oxidant conditions (Livingstone 2001; Machella et al. 2004; Regoli and Principato 1995; Regoli et al. 2004). The possibility of detecting loss of DNA integrity at locations 3, 4, and 5 is certainly useful for a better assessment of toxicologic risks associated with atmospheric pollutants.

In the present study, the ecotoxicologic approach appears to be a valuable tool for monitoring air quality in urban areas. The snail *H. aspersa* was an efficient bioindicator that accumulated bioavailable contaminants and allowed the integration of these data with toxicologic responses. Results obtained in the urban area of Ancona indicate that vehicular traffic plays a prominent role in the perturbed responses of *H. aspersa*, suggesting the potential use of land snails in larger monitoring networks. These snails might also be used to evaluate the efficacy of mitigation decisions or temporary or long-term variations of atmospheric pollutants. This pilot study suggests that important ramifications need to be explored. It is unknown whether the response of sentinel species to urban pollutants can be influenced by natural fluctuations in biologic features (e.g., metabolic status and reproductive cycle), by the seasonality of environmental factors (e.g., traffic intensities and emissions, temperature, or raining regimes), or the local characteristics of different urban areas.

The ecotoxicologic approach described here might also have a relevance for the impact of atmospheric pollutants on both ecosystems and human health. Snails are representative primary consumers in terrestrial food webs and can thus be important indicators of the potential transfer of pollutants to higher trophic levels (Gomot de Vaufleury and Pihan 2000). Deleterious health effects caused by airborne chemicals have been widely documented in humans (Maynard 2004), although only a few studies have attempted to relate human disease incidence with biomonitoring outcomes (Cislaghi and Nimis 1997; Wappelhorst et al. 2000). Both homologies and differences in toxicologic responses can be expected between snails and human models. Oxidative mechanisms and pollutant-mediated ROS generation are well recognized in humans and have been related to epidemiologic evidence and deleterious health effects caused by vehicular traffic in several urban areas (Sioutas et al. 2005). Other cellular pathways, such as peroxisomal proliferation and biotransformation of PAHs by cytochrome P450, are more responsive in humans, enhancing the carcinogenic properties of aromatic chemicals. Although in our study snails were presumably exposed to particles of respirable size, the link between observed responses and human health link would be strengthened by some direct analyses of air pollutants and a better assessment of exposure profiles in individuals with different lifestyles (specific jobs, time spent in or near vehicles, location of working and living places, etc.). At present, it is difficult to address the transferability of our results obtained in gastropods to expected thresholds/effects in humans or ecologic populations. However, this uncertainty should stimulate the development of multi-disciplinary programs integrating emission control and analytical monitoring of air samples, use of sentinel species, laboratory investigations and toxicity tests, and ecologic and epidemiologic studies.

## Figures and Tables

**Table 1:** Concentrations (mean ± SD) of trace metals and PAHs (μg/g dry weight) in the digestive gland of snails caged in various urban sites (*n* = 5/group).

Contaminant	*p*-Value	Site 1	Site 2	Site 3	Site 4	Site 5
Metals						
Cd	NS	5.61 ± 0.93	8.60 ± 2.04	5.60 ± 1.58	7.29 ± 2.12	8.93 ± 2.22
Cr	*p* < 0.001	0.35 ± 0.05	0.57 ± 0.03	1.30 ± 0.68*	1.25 ± 0.63*	1.65 ± 0.66*
Cu	*p* < 0.0005	8.65 ± 1.34	17.4 ± 2.12*	21.2 ± 1.91*	80.8 ± 22.9**	75.0 ± 26.3**
Fe	*p* < 0.0001	87.5 ± 8.04	150 ± 25.5*	2,016 ± 886#	959 ± 478**	555 ± 273**
Pb	*p* < 0.0001	1.62 ± 0.35	5.06 ± 0.88*	3.12 ± 0.92*	15.2 ± 5.22**	80.5 ± 39.5#
Mn	*p* < 0.005	146 ± 17.5	223 ± 31.1*	467 ± 197**	517 ± 209**	409 ± 166**
Ni	*p* < 0.001	0.39 ± 0.08	1.23 ± 0.19*	1.56 ± 0.19*	0.98 ± 0.36*	2.64 ± 1.32*
Zn	*p* < 0.005	126 ± 16.1	183 ± 30.4*	297 ± 91.0**	502 ± 187#	514 ± 199#
PAHs						
Naphthalene	*p* < 0.005	260 ± 73.0	344 ± 19.7*	389 ± 70.0*	497 ± 204**	502 ± 41.8**
Acenaphthene		ND	ND	ND	ND	ND
Fluorene	*p* < 0.01	35.3 ± 11.3	49.9 ± 18.7	64.7 ± 2.13*	56.8 ± 19.0*	63.3 ± 5.38*
Phenanthrene	NS	8.70 ± 3.44	12.4 ± 3.68	15.4 ± 0.71	11.0 ± 3.60	11.9 ± 2.70
Anthracene	*p* < 0.01	0.57 ± 0.26	2.12 ± 0.36*	4.56 ± 1.66*	2.77 ± 1.67*	5.02 ± 0.45*
Fluoranthene	*p* < 0.005	0.59 ± 0.35	0.38 ± 0.31	1.82 ± 1.82	17.8 ± 11.2**	4.24 ± 3.56*
Pyrene	*p* < 0.001	7.39 ± 3.44	7.37 ± 3.88	19.5 ± 3.24*	10.9 ± 9.38*	19.4 ± 9.46*
Benzo[*a*]anthracene		ND	ND	ND	1.08 ± 0.64	3.13 ± 0.44
Chrysene		ND	ND	ND	ND	ND
Benzo[*b*]fluoranthene		ND	ND	ND	2.12	2.66 ± 2.64
Benzo[*k*]fluoranthene		ND	ND	ND	0.66 ± 0.86	1.20 ± 0.40
Benzo[*a*]pyrene		0.85 ± 0.13	ND	ND	ND	ND
Dibenzo[*a,h*]anthracene		ND	ND	ND	ND	ND
Benzo[*g,h,i* ]perylene		ND	ND	ND	ND	ND
Total lmw PAHs	*p* < 0.005	305 ± 82.9	409 ± 33.9*	473 ± 70.1*	568 ± 226**	582 ± 46.6**
Total hmw PAHs	*p* < 0.001	8.54 ± 2.77	7.76 ± 4.19	20.7 ± 4.81*	32.6 ± 4.32*	28.2 ± 8.41*
Total PAHs	*p* < 0.005	314 ± 85.1	417 ± 32.7*	494 ± 72.5*	601 ± 109**	610 ± 53.0**

Abbreviations: ND, not detectable; NS, not significant.

* *p* < 0.05

** *p* < 0.001

# *p* < 0.0001 indicate significant variations and differences between groups of means (post hoc comparison).

**Table 2:** Biochemical and cellular biomarkers in the digestive gland of *H. aspersa* (mean ± SD; *n* = 5/group).

Biomarker	*p*-Value	Site 1	Site 2	Site 3	Site 4	Site 5
MT [eq.(G)SH nmol/mg protein]	*p* < 0.001	3.41 ± 2.14	9.33 ± 3.35*	12.1 ± 1.58*	13.7 ± 3.98**	15.4 ± 5.31**
AOX (nmol/min/mg protein)	*p* < 0.05	0.11 ± 0.05	0.10 ± 0.05	0.48 ± 0.08**	0.26 ± 0.06*	0.26 ± 0.07*
EROD activity (pmol/min/mg protein)	NS	0.62 ± 0.09	0.33 ± 0.10	0.52 ± 0.10	0.38 ± 0.20	0.66 ± 0.13
ECOD activity (pmol/min/mg protein)	*p* < 0.05	1,432 ± 101	1,279 ± 219	2,440 ± 781*	1,682 ± 285	2,163 ± 395*
Catalase (μmol/min/mg protein)	*p* < 0.0005	321 ± 48.7	323 ± 46.2	700 ± 144*	545 ± 51.3*	697 ± 257*
GR (nmol/min/mg protein)	*p* < 0.001	16.1 ± 3.54	12.7 ± 1.27	22.4 ± 5.08*	29.0 ± 7.77*	26.1 ± 5.69*
GST (nmol/min/mg protein)	*p* < 0.05	1,892 ± 219	1,629 ± 555	2,214 ± 363	1,540 ± 371	1,219 ± 125*
GPx (nmol/min/mg protein)	NS	13.1 ± 5.66	12.9 ± 2.51	7.68 ± 4.35	15.3 ± 2.95	19.3 ± 9.17
Total glutathione (μmol/g tissue)	NS	1.63 ± 0.25	1.38 ± 0.42	1.05 ± 0.33	1.60 ± 0.16	1.94 ± 0.49
TOSC (ROO•; U/mg protein)	*p* < 0.01	981 ± 96.1	826 ± 102	1,588 ± 61.7*	1,330 ± 147*	1,437 ± 105*
TOSC (HO•; U/mg protein)	*p* < 0.01	1,071 ± 132	953 ± 259	1,544 ± 189*	1,206 ± 132*	1,470 ± 219*
NRRT (min)	*p* < 0.0005	27.4 ± 0.66	11.6 ± 7.03*	20.4 ± 8.64	9.97 ± 7.28*	10.8 ± 7.62*
DNA TD (arbitrary units)	*p* < 0.005	128 ± 15.6	111 ± 5.28	197 ± 74.9*	184 ± 44.9*	215 ± 64.85*

Abbreviations: eq, equivalents; NS, not significant.

* *p* < 0.05 and

** *p* < 0.001 indicate significant variations and differences between groups of means (post hoc comparison).

**Table 3:** Eigenvalues, percentage, and total variance of factors obtained from PCA analysis of chemical and biologic parameters of the land snail *H. aspersa.*

Axis	Eigen-value	Percent variance	Cumulative variance	Contaminant/biomarker	Axis 1 (PC1)	Axis 2 (PC2)	Axis 3 (PC3)	Axis 4 (PC4)
				Cu	0.790705†	0.539160	0.165378	0.238207
PC1	17.70858	59.02861	59.0286	Pb	0.715212†	0.500997	−0.542411	−0.110842
				Cd	0.313324	0.752549†	−0.005360	−0.579197
PC2	6.91878	23.06260	82.0912	Cr	0.997280†	−0.008272	−0.070683	−0.019186
				Ni	0.784127†	0.126048	−0.399784	−0.457635
PC3	3.02699	10.08994	92.1812	Mn	0.900092†	−0.161097	0.377186	0.147014
				Fe	0.595581	−0.764651†	0.238275	0.061795
PC4	2.34565	7.81885	100.00	Zn	0.917575†	0.341097	0.113295	0.169924
				Naphthalene	0.922264†	0.331423	0.198704	0.010255
				Fluorene	0.920068†	−0.215378	0.108228	−0.308827
				Phenanthrene	0.516094	−0.722838†	0.174026	−0.561636
				Anthracene	0.913881†	−0.204078	−0.170257	−0.306899
				Fluoranthene	0.475363	0.353075	0.626410	0.506930
				Pyrene	0.846901†	−0.382454	−0.355005	−0.102269
				Total lmw PAHs	0.946469†	0.257876	0.190068	−0.039624
				Total hmw PAHs	0.920453†	0.156682	0.158835	0.320918
				Total PAHs	0.949531†	0.250606	0.188443	−0.008797
				MT	0.948667†	0.145167	0.175937	−0.219096
				AOX	0.693021†	−0.716261†	0.065546	0.048953
				EROD activity	0.158404	−0.108723	−0.921801†	0.336705
				ECOD activity	0.786172†	−0.549701	−0.281625	0.021227
				Catalase	0.939616†	−0.299041	−0.163484	0.031132
				GR	0.867874†	0.105793	0.137845	0.465404
				GST	−0.354490	−0.917779†	0.134982	0.117469
				GPx	0.309859	0.901686†	−0.284869	0.098988
				Total glutathione	0.171919	0.840484†	−0.437953	0.268751
				TOSC (ROO•)	0.865488†	−0.434499	−0.099349	0.228628
				TOSC (HO•)	0.821282†	−0.464366	−0.320765	0.083491
				Lysosomal NRRT	−0.555389	−0.556256	−0.400064	0.471244
				DNA TD	0.947211†	−0.181370	−0.170131	0.202365

Abbreviations: PC, principal component; PC1, axis 1; PC2, axis 2; PC3, axis 3; PC4, axis 4. Factor loadings are given for each parameter.

† Values ≥ 0.7.
